# Clinical implications of somatic allele expansion in female FMR1 premutation carriers

**DOI:** 10.1038/s41598-023-33528-x

**Published:** 2023-04-29

**Authors:** Ramkumar Aishworiya, Ye Hyun Hwang, Ellery Santos, Bruce Hayward, Karen Usdin, Blythe Durbin-Johnson, Randi Hagerman, Flora Tassone

**Affiliations:** 1 Medical Investigation of Neurodevelopmental Disorders (MIND) Institute, University of California Davis, 2825 50Th Street, Sacramento, CA 95817 USA; 2 Khoo Teck Puat-National University Children’s Medical Institute, National University Health System, 5 Lower Kent Ridge Road, Singapore, 119074 Singapore; 3 Department of Pediatrics, Yong Loo Lin School of Medicine, National University of Singapore, 10 Medical Drive, Singapore, 117597 Singapore; 4 Department of Biochemistry and Molecular Medicine, University of California Davis, School of Medicine, 4610 X St, Sacramento, CA 95817 USA; 5 Department of Pediatrics, University of California Davis, School of Medicine, 4610 X St, Sacramento, CA 95817 USA; 6 Laboratory of Cell and Molecular Biology, Digestive and Kidney Diseases, National Institute of Diabetes, 9000 Rockville Pike, Bethesda, MD 20892 USA; 7 Department of Public Health Sciences, University of California, Davis, School of Medicine, 4610 X St, Sacramento, CA 95817 USA

**Keywords:** Disease genetics, ADHD

## Abstract

Carriers of a premutation allele (PM) in the *FMR1* gene are at risk of developing a number of Fragile X premutation asssociated disorders (FXPAC), including Fragile X-associated Tremor/Ataxia Syndrome (FXTAS), Fragile X-associated Primary Ovarian Insufficiency (FXPOI), and Fragile X-associated neuropsychiatric disorders (FXAND). We have recently reported somatic CGG allele expansion in female PM; however, its clinical significance remains unclear. The aim of this study was to examine the potential clinical association between somatic *FMR1* allele instability and PM associated disorders. Participants comprised of 424 female PM carriers age 0.3– 90 years. *FMR1* molecular measures and clinical information on the presence of medical conditions, were determined for all subjects for primary analysis. Two sub-groups of participants (age ≥ 25, N = 377 and age ≥ 50, N = 134) were used in the analysis related to presence of FXPOI and FXTAS, respectively. Among all participants (N = 424), the degree of instability (expansion) was significantly higher (median 2.5 vs 2.0, *P* = 0.026) in participants with a diagnosis of attention deficit hyperactivity disorder (ADHD) compared to those without. *FMR1 mRNA* expression was significantly higher in subjects with any psychiatric disorder diagnosis (*P* = 0.0017); specifically, in those with ADHD (*P* = 0.009), and with depression (*P* = 0.025). Somatic *FMR1* expansion was associated with the presence of ADHD in female PM and *FMR1* mRNA levels were associated with the presence of mental health disorders. The findings of our research are innovative as they suggest a potential role of the CGG expansion in the clinical phenotype of PM and may potentially guide clinical prognosis and management.

## Introduction

The fragile X premutation carrier status has received growing recognition as a distinct clinical and molecular entity beyond Fragile X syndrome (FXS) over the past two decades. PM individuals have between 55 to 200 CGG repeats in the 5’ region of the *FMR1* gene and typically pass on the mutation to their offspring, with a propensity for expansion to the full mutation state (> 200 CGG repeats) in each subsequent generation^1^. While FXS is one of the most common single gene disorders causing intellectual disability, the premutation status is more common and occurs in approximately 1 in 400 males and 1 in 200 females^2^. We now know that the premutation status is associated with specific clinical conditions including Fragile X-associated Tremor/Ataxia Syndrome (FXTAS), Fragile X-associated Primary Ovarian Insufficiency (FXPOI), and Fragile X-associated Neuropsychiatric Disorders (FXAND)^3–5^. Other research groups have referred to these neuropsychiatric and other conditions associated with the premutation as Fragile X-premutation associated conditions (FXPAC) due to concerns of stigma associated with the term ‘disorder’. Regardless, it is important to recognize the presence and burden of these conditions in PM carriers as many of them are associated with significant comorbidity.

Occurrence of these disorders varies across PM with gender differences as well. For instance, FXTAS is seen in 40–75% of male PM and 8–16% of females^6,7^. Common FXAND conditions include anxiety (occurring in 50–70% of carriers) and depression (40–65% of carriers) but increasingly, attention deficit hyperactivity disorder (ADHD) and autism spectrum disorder (ASD) have also been reported to be more common in PM, with reported prevalence rates of 30–45% and 8% respectively^8–12^. The molecular basis behind these disorders is attributed to the toxicity related to elevated levels of *FMR1* mRNA seen in PM^13,14^. This is proposed to interfere with various cellular functions through mitochondrial dysfunction, calcium dysregulation and cell/DNA damage repair functions^13,15–17^.

Instability of the *FMR1* premutation allele has recently been described for the first time by our group on a large sample of female PM^18^. Specifically, we characterized the presence of a series of unstable expanded alleles in ~ 94% of peripheral blood samples differing from the major premutation allele by one or more repeat units. The degree of expansion was directly proportional to the number of CGG repeats in the original allele with an indication that the extent of the expansion increased with age as well. Expansion was also inversely proportional to the number of AGG anchors which are considered to be ‘stabilizers’ that prevent CGG expansion across generations. However, the clinical implications of the presence and degree of this somatic expansion is, as yet, unclear. Indeed, given that this observation was based on peripheral blood sample, how this translates to brain tissue and its possible implications especially in the pathophysiology of neurologic premutation associated conditions like FXAND and FXTAS are important areas for further studies.

The aim of this study was to examine the potential clinical associations between somatic expansion in female PM and premutation associated conditions. We hypothesized that a higher degree of expansion will be associated with a greater occurrence of disorders; and hence planned for separate sub-group analysis by age-range of the cohort a priori.

## Methods

### Subjects

Participants in this study included 424 female PM age 0.3–90 years whose premutation status (CGG repeat size 55–200) was confirmed by PCR/Southern blot testing. *FMR1* molecular measures and clinical information, were determined for all 424 subjects and used in the primary analysis. In addition, participants were divided in two additional sub-groups for the analysis related to the presence of FXPOI and FXTAS (age ≥ 25, N = 377, and age ≥ 50, N = 134 respectively).

Participants were enrolled either as part of a dedicated research visit or following cascade testing after consultation for a child or sibling with fragile X syndrome. The study and all research protocols were carried out in accordance with and was approved by the Institutional Review Board at the University of California, Davis. All participants gave written informed consent before participating in the study.

### Clinical information

Clinical information on the presence and severity of FXTAS, presence of FXPOI, presence of FXAND disorders, neurological symptoms, chronic medical problems and auto-immune conditions and demographic variables including weight, height, history of smoking and education level were documented on a data sheet filled out in taking the medical history. Ascertainment of presence and stage (range 1–5) of FXTAS was made by an experienced physician (RJH) who has several years of experience with PM patients, following detailed medical examination and review of Magnetic Resonance Imaging images of patients. Presence of FXPOI was defined as occurrence of early menopause prior to 40 years of age.

Presence of FXAND disorders was determined based on DSM-5 criteria for respective FXAND conditions, following evaluation by a trained medical provider or psychologist. FXAND disorders examined, specifically included anxiety, depression, ASD and ADHD and standardized assessments such as the Autism Diagnostic Observation Schedule (ADOS)^19^, the Structured Clinical Interview for DSM-5 (SCID-5)^20^, and the Kiddie Schedule for Affective Disorders and Schizophrenia (K-SADS)^21^, for younger patients, were used. Information on results of the SCID-5 In terms of presence of at least 1 psychiatric disorder (e.g. specific phobias, social phobia, generalized anxiety disorder, major depressive disorder) was obtained. A cognitive assessment based on standardized testing was carried out. Given the wide age-range of participants, a variety of standardized cognitive assessments were used across study participants. These included the Wechsler Intelligence Scale for Children, Third or Fourth Edition (WISC-III or WISC-IV)^22^, the Stanford Binet Intelligence Scales, Fifth Edition (SB-5)^23^, and the Wechsler Adult Intelligence Scale, Third or Fourth Edition (WAIS-III or WAIS-IV)^24^, as per participant age.

Specific chronic medical conditions of interest comprised hyper or hypothyroidism, diabetes mellitus, migraine, osteoporosis, seizures and autonomic dysfunction such as urinary incontinence. Information on these conditions were collapsed into a single category titled chronic medical conditions with presence of at least 1 condition and number of conditions collected. Specific auto-immune conditions included systemic lupus erythematosus, rheumatoid arthritis, fibromyalgia, autoimmune thyroid conditions and multiple sclerosis. Similarly, presence of at least 1 auto-immune condition and number of such conditions was collected under the category of auto-immune conditions.

### Molecular data

Molecular data, including CGG allele repeat size, AGG interruptions and expansion were obtained from DNA samples isolated from 3 mL of whole blood using the Gentra Puregene Blood Kit (Qiagen, Valencia, CA, USA). *FMR1* allele size was assessed through PCR and Southern blot analysis as previously described^25,26^. PCR used specific *FMR1 primers* (AmplideX PCR/CE, Asuragen) and PCR products were visualized by capillary electrophoresis (CE). Southern blot analysis was used to determine the methylation status of the *FMR1* alleles (Activation ratio, AR and percent of methylation) as reported in Tassone et al.^27^ The AR value indicates the percentage of cells carrying the normal allele on the active X-chromosome**.** The number of AGG interruptions were quantified with a triplet primed PCR protocol as reported in Yrigollen et al.^28,29^, which was then visualized with CE and analyzed with Peak Scanner Software 2.0. To quantify the degree of expansion in each participant, the original size allele (Peak 1) and the modal expanded allele (Peak 2) needed to be identified, in which identification of Peak 1 was facilitated by X-inactivation in females. The expanded peak with the highest relative fluorescence units (RFU) value was selected as Peak 2. Expansion was calculated using the formula (Peak 2–Peak 1), more details can be found in Hwang et al.^18^. Total RNA was isolated from 2.5 ml of peripheral blood collected in PAXgene (Qiagen, Valencia, CA, United States) and quantified using the Agilent 2100 Bioanalyzer system. To measure *FMR1* transcript expression levels, qRT-PCR used Assays-On-Demand (Applied Biosystems, Foster City, CA, USA) and custom TaqMan primers and probe assays as specified by Tassone et al.^13^.

### Statistical analysis

All analysis was conducted using R version 4.0.5 (2021-03-31). Analysis was conducted on all participants (0.3–90 years old) as well as on those age ≥ 25 years (FXPOI group) and those ≥ 50 years (FXTAS group) as separate sub-groups. Specific measures analyzed in the FXPOI group was the age at menopause and the presence of FXPOI while that in the FXTAS group was the presence and the stage of FXTAS. Expansion was measured as described in our previous paper^18^ but in essence, instability was represented by ∆Rpts, which, in a repeat profile, represents the difference in the number of repeats between the modal expanded allele (Peak 2) and modal stable allele (Peak 1). Spearman test of correlation were used to examine CGG repeat length, *FMR1* mRNA expression, and expansion (measured as peak 2—peak 1) against IQ measures. CGG repeat length, *FMR1* mRNA expression, and expansion, were compared between subjects with and without a given clinical condition using Wilcoxon rank sum tests. *FMR1* was modelled by CGG, adjusting for activation ratio (AR), using a linear regression model with *FMR1* as the response and CGG and AR as covariates. Analyses of associations between expansion and clinical phenotype was not adjusted for CGG repeat number as none of the clinical conditions were significantly associated with the number of CGG repeats and CGG repeats furthermore did not differ meaningfully between subjects with and without a given condition.

### Ethics approval and consent to participate

The study and all research protocols were carried out in accordance with the Institutional Review Board at the University of California, Davis. All participants gave written informed consent before participating in the study.

## Results

**Figure 1:**
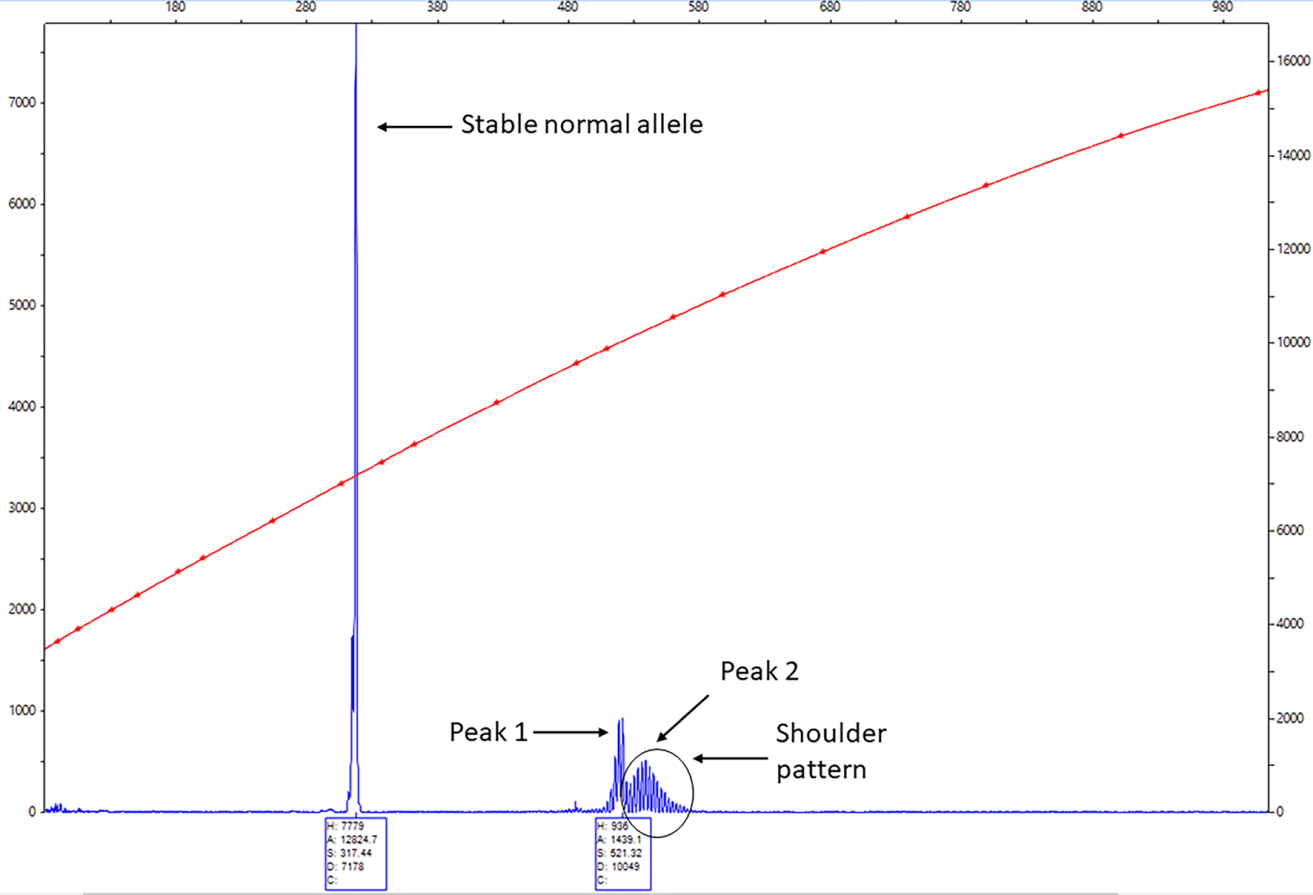
Example of a PCR profile (visualized by a Capillary Electrophoretogram) observed in a female PM showing somatic allelic instability. The degree of CGG instability is shown as a shoulder pattern (serial of peaks) located to the right side of the premutation allele peak. Instability is calculated as Peak 2 (unstable premutation allele size, highest peak size of the shoulder pattern) minus Peak 1 (stable premutation allele size on the left of the shoulder pattern) as described in Hwang et al. 2022.

The final number of participants consisted of 424 female PM with an age range of 0.3 to 90 years. The mean number of CGG repeats was 92.1 (SD 23.0); premutation expansion, was present in all 424 participants and somatic instability, as illustrated in Fig. 1, was present in ~ 92% of them. Average full-scale IQ of participants was 119.8 (SD 19.0). Table 1 shows the descriptive molecular and clinical data of the three groups of participants. There was a large range of clinical conditions present within participants with the most common ones being anxiety (77.3% of those who had available data) and depression (63.6%). The majority of the participants (84.0%) had at least one FXAND condition. Among the 43 participants who had FXPOI within the $\ge$ 25 years old sub-group, 36 (85.7%) had anxiety, 30 (71.4%) had depression, 13 (30.2%) had autoimmune conditions.

**Table 1:** Molecular and clinical data of participants included in the study.

	All Subjects (n = 424)			FXPOI≥ 25 years (n = 377)			FXTAS, ≥ 50 years (n = 134)		
*Molecular Measures*	n	Mean (SD)	Median (Range)	n	Mean (SD)	Median (Range)	n	Mean (SD)	Median (Range)
*Age*	422	42.5 (17.2)	41 (0.3–90)	375	46.2 (14.2)	42 (25–90)	132	62.6 (9.4)	61 (50–90)
*CGG*	423	91.8 (22)	89 (55–196)	376	91.7 (20)	89.5 (55–190)	134	88 (19.3)	87 (55–161)
*FMR1 mRNA*	400	2.2 (0.9)	2.1 (0–7)	359	2.2 (0.9)	2.1 (0–7)	124	1.9 (0.9)	2 (0–7)
*Instability*	410	3.3 (6.1)	2 (0–56)	366	3.2 (5.9)	2 (0–56)	127	3.4 (5.9)	2 (0–56)
*AGG*	424	0.8 (0.8)	1 (0–2)	377	0.8 (0.8)	1 (0–2)	134	0.8 (0.8)	1 (0–2)
*0*		195 (46%)			172 (45.6%)			62 (46.3%)	
*1*		138 (32.5%)			125 (33.2%)			42 (31.3%)	
*2*		91 (21.5%)			80 (21.2%)			30 (22.4%)	

	All Subjects (n = 424)			FXPOI, ≥ 25 years (n = 377)			FXTAS, ≥ 50 years (n = 134)		
*Clinical Measures*	n	No	Yes	n	No	Yes	n	No	Yes
*Medical conditions*	187	62 (33.2%)	125 (66.8%)	180	58 (32.2%)	122 (67.8%)	93	20 (21.5%)	73 (78.5%)
*Autoimmune conditions*	187	145 (77.5%)	42 (22.5%)	180	138 (76.7%)	42 (23.3%)	93	66 (71%)	27 (29%)
*SCID diagnosis*	97	22 (22.7%)	75 (77.3%)	81	20 (24.7%)	61 (75.3%)	18	4 (22.2%)	14 (77.8%)
*Autism*	174	170 (97.7%)	4 (2.3%)	–	–	–	–	–	–
*ADHD*	187	138 (73.8%)	49 (26.2%)	180	133 (73.9%)	47 (26.1%)	93	73 (78.5%)	20 (21.5%)
*Anxiety*	181	41 (22.7%)	140 (77.3%)	175	40 (22.9%)	135 (77.1%)	91	23 (25.3%)	68 (74.7%)
*Depression*	184	67 (36.4%)	117 (63.6%)	177	64 (36.2%)	113 (63.8%)	90	29 (32.2%)	61 (67.8%)
*Osteoporosis*	141	122 (86.5%)	19 (13.5%)	134	115 (85.8%)	19 (14.2%)	76	58 (76.3%)	18 (23.7%)
*FXAND*	188	30 (16%)	158 (84%)	181	30 (16.6%)	151 (83.4%)	93	17 (18.3%)	76 (81.7%)
*FXTAS Diagnosis*	–	–	–	–	–	–	77	26 (33.8%)	51 (66.2%)
*FXPOI*	–	–	–	132	89 (67.4%)	43 (32.6%)	77	49 (63.6%)	28 (36.4%)

	n	Mean (SD)	Median (Range)	n	Mean (SD)	Median (Range)	n	Mean (SD)	Median (Range)
*Full Scale IQ*	122	119.8 (19)	118 (71–161)	122	119.8 (19)	118 (71–161)	70	116.3 (18.8)	112.5 (71–157)
*Verbal IQ*	123	97.2 (20.9)	103 (48–134)	123	97.2 (20.9)	103 (48–134)	70	94.3 (22.5)	102 (48–134)
*Performance IQ*	123	96.4 (27.1)	104 (31–150)	123	96.4 (27.1)	104 (31–150)	70	87.6 (26.3)	94 (31–138)
	Total Subjects (n = 92)								
*Smoking*	n	Percentage		–	–	–	–	–	–
*Current Smoker*	5	5 (5.4%)	–	–	–	–	–	–	–
*Former Smoker*	30	30 (32.6%)				–	–	–	–
*Never Smoker*	57	57 (62%)	–	–	–	–	–	–	–
	Total Subjects (n = 111)								
*Education*	n	Percentage							
*Less Than High School*	4	3.60%	–	–	–	–	–	–	–
*High School/GED*	21	18.90%				–	–	–	–
*Partial College*	27	24.30%	–	–	–	–	–	–	–
*College or Higher*	59	53.20%	–	–	–	–	–	–	–

**Figure 2:**
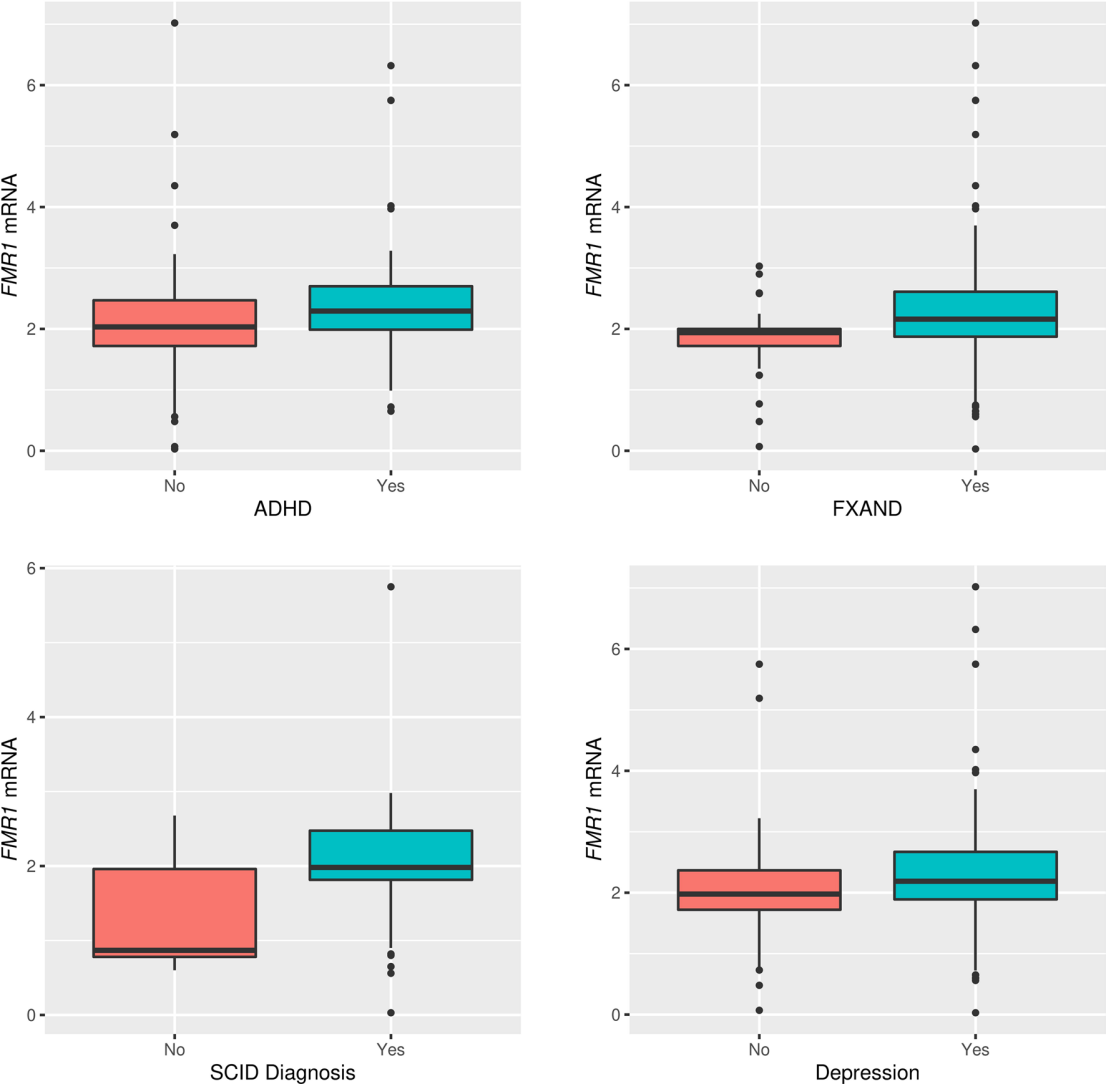
Significant association between *FMR1* mRNA levels and the presence of clinical conditions.

Looking at the entire cohort, the degree of expansion was significantly higher in participants with a diagnosis of ADHD (median difference 2.5) compared to those without the diagnosis (median difference 2.0, *P* = 0.026) (Table 2). A sensitivity analyses of the association between instability and ADHD, adjusting for age, yielded the same conclusions as the unadjusted analysis. Expansion was not associated with any other clinical condition. *FMR1* mRNA expression was significantly higher in participants with at least 1 SCID-5 diagnosis (median difference 1.98 vs 0.87, *P* = 0.0017), in those with ADHD (2.30 vs 2.04, *P* = 0.009), in those with depression (2.19 vs 1.98, *P* = 0.025) and in those with at least 1 FXAND condition (2.16 vs 1.94, *P* = 0.005). (Table 3 and Fig. 2). Full-scale IQ was not significantly correlated with either expansion, *FMR1* mRNA or number of CGG repeats*.*

**Table 2:** Expansion by clinical conditions.

	Median (25%, 75%) without conditions	Median (25%, 75%) with conditions	*P*-value
*Expansion by clinical conditions*			
FXAND	2 (0, 4)	2 (0, 5)	0.612
Medical conditions	2 (0, 4)	2 (0, 5)	0.703
Autoimmune conditions	2 (0, 4.25)	1 (0, 5)	0.207
SCID diagnosis	2 (0, 7)	2 (0, 4)	0.246
Autism	2 (0, 4.5)	2 (1, 2.5)	0.718
Anxiety	3 (0, 5)	2 (0, 4)	0.228
ADHD	2 (0, 3.5)	2.5 (0.25, 6)	**0.026**
Depression	2 (0, 4)	2 (0, 5)	0.497
Osteoporosis	2 (0, 4)	0 (0, 6)	0.671

^a^*P*-values are from Wilcoxon rank-sum tests; FXAND: Fragile X- associated neurodevelopmental disorders, SCID: Structured Clinical Interview for DSM-5, ADHD: attention deficit hyperactivity disorder.

**Table 3:** FMR1 mRNA by clinical conditions.

	Median (25%, 75%) without conditions	Median (25%, 75%) with conditions	*P*-value
*FMR1 mRNA by clinical conditions*			
FXAND	1.94 (1.72, 2)	2.16 (1.87, 2.61)	**0.0045**
Medical conditions	2.3 (1.92, 2.68)	2.06 (1.75, 2.48)	0.066
Autoimmune conditions	2.095 (1.81, 2.58)	2.14 (1.89, 2.47)	0.782
SCID diagnosis	0.87 (0.78, 1.96)	1.98 (1.81, 2.47)	**0.0017**
Autism	2.1 (1.84, 2.56)	2.065 (1.90, 2.92)	0.859
Anxiety	1.96 (1.74, 2.60)	2.155 (1.88, 2.56)	0.395
ADHD	2.035 (1.72, 2.47)	2.295 (1.99, 2.7)	**0.0085**
Depression	1.975 (1.72, 2.37)	2.19 (1.89, 2.67)	**0.024**
Osteoporosis	2.06 (1.84, 2.47)	1.95 (1.53, 2.41)	0.65

^a^*P*-values are from Wilcoxon rank-sum tests; FXAND: Fragile X associated neurodevelopmental disorders, SCID: Structured Clinical Interview for DSM-5, ADHD: attention deficit hyperactivity disorder.

**Figure 3:**
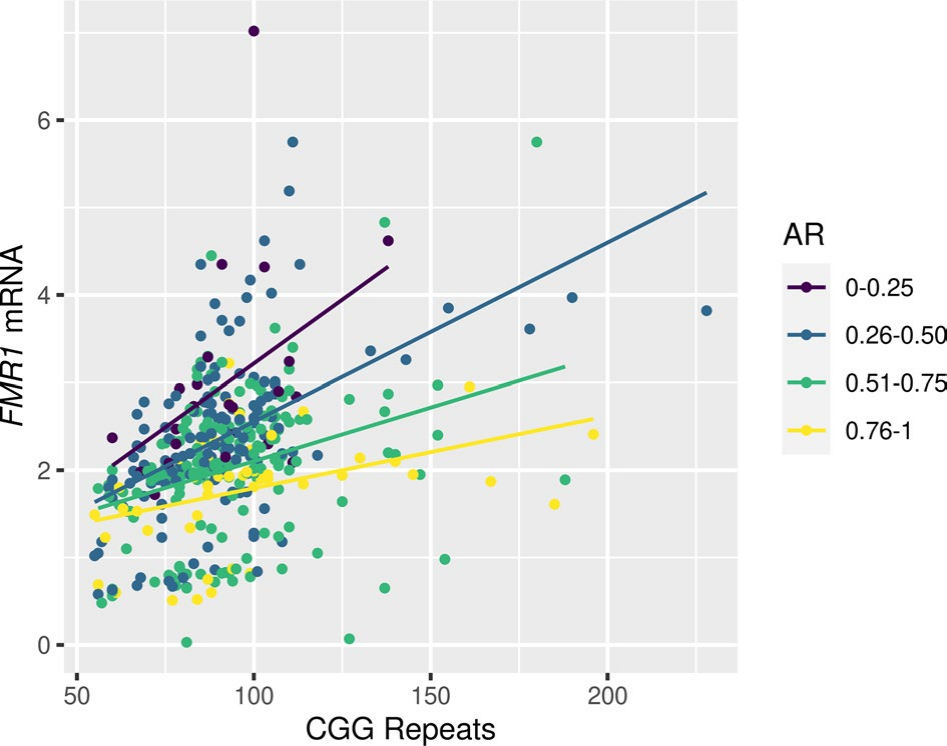
Scatterplot of correlation between *FMR1* mRNA levels by CGG repeat number, stratified by activation ratio.

Analysis was also done to look at the presence of clinical conditions by other molecular measures namely number of CGG repeat numbers, number of AGG anchors and the AR. CGG repeat length did not differ significantly with presence of any clinical condition. However, among those with > 105 CGG repeats, there was a greater proportion of participants with ADHD compared to those with < 105 CGG repeats; although this was not statistically significant. The proportion of participants with ADHD was also highest in those with an AR between 0.21 and 0.40 as compared to other ranges. There were no other significant associations with CGG repeat numbers or AR for any clinical condition. Lastly, although in our analysis we observed differences in the number of AGG anchors, they did not reach significance with the presence of any clinical condition. As expected, after adjusting for AR, *FMR1* mRNA levels increased significantly with increasing number of CGG repeats (B = 0.016, SE = 0.002, *P* =  < 0.001) (Fig. 3). Further, the increase in levels of *FMR1* mRNA was clearly modulated by the AR, with participants who had a higher ratio having lower increase in *FMR1* mRNA levels.

Looking at demographic variables associated with clinical conditions, participants who had ADHD were significantly younger than those without the condition, as expected (mean age 47.0 years vs 52.0; *P* = 0.037). Those who had anxiety were much more likely to have a lower educational status as compared to those who did not have anxiety (47.0% with college education or higher vs 75.0%, *P* = 0.034). Age, educational attainment, or history of smoking were not associated with any other clinical condition.

**Figure 4:**
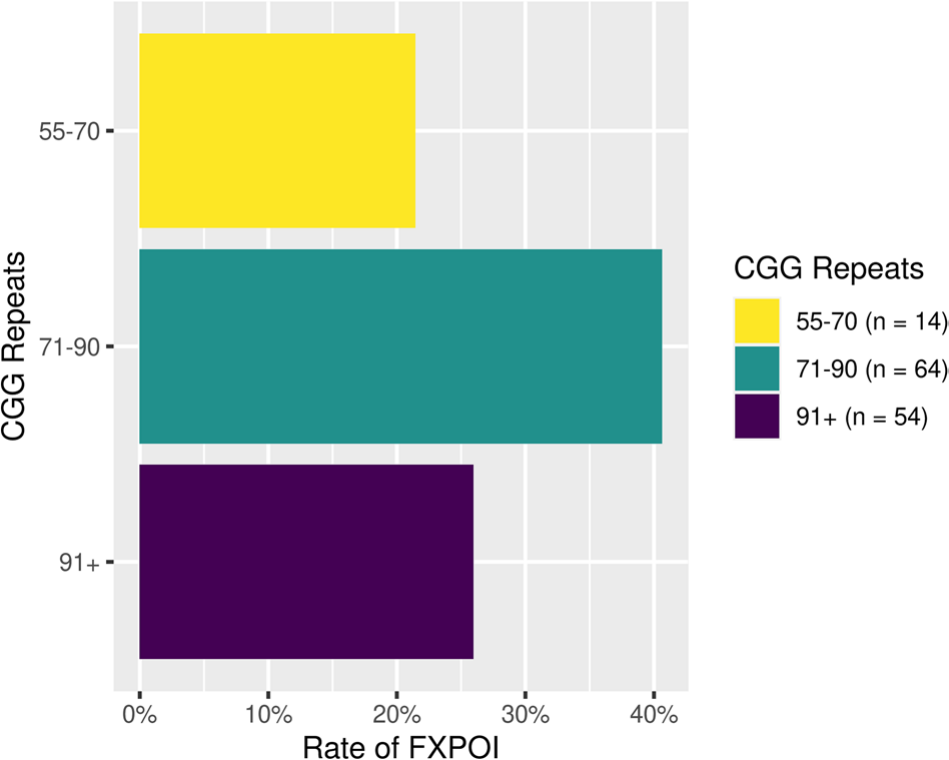
Association between rate of FXPOI and CGG repeat number.

Among the FXPOI sub-group, there was no significant association noted between presence of FXPOI and degree of expansion (median expansion in those with FXPOI vs those without FXPOI 2.0 vs 2.0, *P* = 0.875). The rate of FXPOI was associated with number of CGG repeats as shown in the bar plots in Fig. 4) with the highest prevalence observed in those carrying an allele in the mid-premutation range as previously reported^30^. Furthermore, CGG repeat length was significantly larger (median 3.0 vs 2.0, *P* = 0.020) and allelic expansion higher in subjects with ADHD (median 92.5 vs 87.0, *P* = 0.027). In the FXTAS sub-group, expansion was not associated with the presence or the stage of FXTAS. Interestingly, degree of expansion was lower in subjects with at least 1 diagnosis on the SCID-5 (0.0 vs 2.5, *P* = 0.0428), although this was based on a small number of subjects (N = 18).

## Discussion

This study sought to assess the clinical implications, if any, of somatic allelic expansion in a large cohort of female PM participants. Our main findings suggest that expansion is associated with significantly higher occurrence of ADHD among female PM. Further, higher levels of *FMR1* mRNA are associated with an occurrence of neuropsychiatric conditions including depression and ADHD. Lastly, levels of *FMR1* mRNA were significantly predicted by number of CGG repeats and by AR amongst female PM, confirming findings from previous studies^31–33^.

Our findings with respect to *FMR1* mRNA levels likely reflect the toxicity of elevated levels of mRNA, which has been shown to interfere with mitochondrial function, calcium regulation and cellular repair mechanisms as well as contributing to gain of function mutations^13,15–17^. These disruptions at the molecular level are thought to contribute to the increased pathology seen in PM, resulting in white matter disease in those with FXTAS and other psychiatric problems associated with FXAND. The positive association between *FMR1* mRNA levels and number of CGG repeats is not surprising and is consistent with previous literature^13^. Elevated levels of *FMR1* mRNA have also been shown to be associated with increased levels of psychological symptoms previously^31,34^; our findings add to this in terms of specific psychiatric conditions associated with mRNA levels. We further demonstrate here the crucial role played by the AR as the more favorable the ratio, with greater proportion of the non-mutated allele being active, the lower the excess mRNA produced.

It has also been shown that PM carriers who have higher repeat numbers (> 120 CGG) tend to have lower FMRP levels as well, likely due to the inefficiency of the translational process^35,36^. This combination of elevated mRNA levels and lowered FMRP levels could create a ‘double-hit’ phenomenon resulting in greater occurrence of disorders associated with both the reduced FMRP expression because translation inefficiency and RNA toxicity due to the presence of elevated levels of *FMR1* mRNA^37^. Indeed, a series of cases including PM with CGG repeats in the upper premutation range has previously demonstrated a high occurrence of affective mood disorders, anxiety and psychotic thinking^37^. The combined pathology resultant of both increased mRNA and lowered FMRP is thought to contribute to this occurrence.

ADHD is a common neurodevelopmental disorder that seen in children and adolescents with a population prevalence rate of typically around 5.0% to 9.8%^38–40^. Elevated levels of ADHD have been demonstrated in PM in several studies^10,12,41,42^. Consistent with these reports, in our cohort of 424 participants, we observed that 26.2% of carriers had a diagnosis of ADHD; and this was more likely in younger participants in keeping with ADHD traditionally being considered a predominantly childhood neurodevelopmental disorder and limited awareness and diagnosis of ADHD in adults in the past. Although the exact reasons for elevated rates of ADHD in PM are not yet clear, neuro-imaging of PM has demonstrated changes in grey matter voxel density in the amygdala, insula and caudate and this may be related to executive function challenges^43^. Furthermore, the increased vulnerability of PM neurons related to mRNA toxicity as discussed above is possibly related to attentional and emotional regulation difficulties^15^. The presence of expansion could be a further modulator of this effect within the nervous system. Importantly, given what is seen in a mouse model of the FXDs^44^, and in patients with other repeat expansion diseases^45,46^, the degree of expansion may be higher in the brain compared to peripheral blood from which this study’s samples were obtained. As we previously showed, given that expansion increases with the number of CGG repeats and that the latter correlates with *FMR1* mRNA levels, the presence of expansion could be a marker for elevated mRNA related toxicity. This may explain the greater occurrence of ADHD within our cohort. However, one of the limitation of our study is that our data was restricted in terms of subtypes of severity and ADHD (inattentive vs hyperactive-impulsive). Thus, future research into the implications of expansion on the nature of ADHD is warranted to shed light on possible associative mechanisms. Further, this study includes a small sample size especially for some for the clinical conditions investigated at various age points, which was related to data availability in medical record review. Consequently, the study may have had limited statistical power for detection of significant associations. Increasing the overall sample size and targeted recruitment of PM of specific age groups could help overcome this in future studies. In addition, participants included were not a population sample but rather a mixture of PM seen for clinical concerns and identified by cascade testing. Hence prevalence of clinical conditions such as FXAND conditions could be higher than true prevalence values. However, this preliminary data suggest a potential role of allelic somatic expansion as a biomarker that may be useful for assessing prognosis and clinical management of patients PM.

## Conclusion

Somatic *FMR1* allele expansion was associated with the presence of ADHD in female PM. In addition, higher *FMR1* mRNA levels were associated with the presence of multiple neuropsychiatric conditions as well as increased number of CGG repeats. As such, this work is of relevance as it indicates that somatic allelic CGG expansion could be a potential molecular marker for the manifestation of neuropsychiatric conditions in PM. It is, therefore, original, as it constitutes the first study where somatic instability of the *FMR1* CGG repeat has been associated with clinical conditions in a large cohort of female PM. Further research on the clinical implications of expansion could potentially help to discern the clinical phenotype and guide prognostication and management of these conditions in these individuals.

## Data Availability

Data can be made available on reasonable request to the corresponding author.

## Abbreviations

PM: Premutation
ADHD: Attention deficit hyperactivity disorder
FXTAS: Fragile X-associated Tremor/Ataxia Syndrome
FXPOI: Fragile X-associated Primary Ovarian Insufficiency
FXAND: Fragile X-associated Neuropsychiatric Disorders
ASD: Autism spectrum disorder
CE: Capillary electrophoresis
RFU: Relative fluorescence units
